# Isolation and characterization of avian coronavirus from healthy Eclectus parrots (*Eclectus roratus*) from Indonesia

**DOI:** 10.14202/vetworld.2019.1797-1805

**Published:** 2019-11-19

**Authors:** G. K. Suryaman, R. D. Soejoedono, A. Setiyono, O. N. Poetri, E. Handharyani

**Affiliations:** 1 Department of Clinic Reproduction and Pathology, Faculty of Veterinary Medicine, Bogor Agricultural University, Jl. Agatis, Kampus IPB, Dramaga, Bogor 16680, Indonesia; 2 Department of Animal Disease and Veterinary Public Health, Faculty of Veterinary Medicine, Bogor Agricultural University, Jl. Agatis, Kampus IPB, Dramaga, Bogor 16680, Indonesia

**Keywords:** Avian coronavirus, Eclectus parrot, Infectious bronchitis

## Abstract

**Background and Aim:**

Avian coronavirus has a wide range of hosts, from chickens and turkeys to wild birds. This virus causes an economically and, possibly, environmentally, important loss in the poultry industry. Therefore, research into the avian coronavirus in various species of birds is required. The Eclectus parrot (*Eclectus roratus*) is an endemic bird to Indonesia and Northern Australia and often kept as pets. At present, there has been limited information about avian coronavirus infection among birds. This study aimed to determine the presence of and to characterize avian coronavirus isolated from Eclectus parrots in Indonesia.

**Materials and Methods:**

Cloacal swab samples were taken from 10 healthy Eclectus parrots (*E. roratus*). Each isolate was propagated into specific pathogen-free embryonated chicken eggs. The presence of avian coronavirus was determined using three sets of primers targeting the 3’ untranslated region (3’-UTR) of avian coronavirus (UTR41+/11−), the N gene of the infectious bronchitis virus (IBVN+/−), and the S1 gene of the IBV (XCE2+/XCE2−). The infectious bronchitis vaccine strain H120 was used as a positive control. Resulting positive bands were sequenced for the S1 gene.

**Results:**

None of the isolates was positive for the 3’-UTR, four isolates were positive for the N gene of infectious bronchitis, and two isolates were positive for the S1 gene of the IBV. However, only one isolate (parrot/Indonesia/BX9/16) was sequenced for the partial S1 gene with primers XCE2+/XCE2−. The partial nucleotide sequence of this isolate showed 100% homology with the IBV GI-13 lineage, specifically with a field isolate of the 4/91 variant 1 Israel and the 4/91 vaccine on the hypervariable region 3 site of the S1 gene.

**Conclusion:**

An IB-like avian coronavirus was isolated from healthy Eclectus parrots. Our results indicate that IBV has a wide range of hosts, which prompt the need to understand the interspecies connection of this virus better.

## Introduction

Avian coronavirus is the main representative of genus *Gammacoronavirus*, family *Coronaviridae*, and order *Nidovirales* [1]. Within the avian coronavirus group, the infectious bronchitis virus (IBV) is among the most researched. This virus is known to cause an important disease that incurs a high economic loss in the poultry industry [2,3] despite an ongoing vaccination program. It causes respiratory disease while also affecting the kidneys and reproductive tract [4] through viremia with a severity that differs depending on serotypes [3]. Mutations and recombination have produced a high genetic diversity of the virus. In addition, vaccinations performed in the farm setting can influence the evolution of the virus [5]. Many serotypes of IBV are often not cross-protective [6,7]. Mismatching between the circulating strain and the administered vaccine may contribute to vaccination failure [7]. Ubiquitous IBV and IBV-like viruses have also been found in species other than chicken, such as in peafowl, guinea fowl, partridge, waterfowl, and teal [2,6,8-10]. This finding strengthens the possibility that IBV may have a wider range of hosts than previously thought [8,6]. Despite this, data relating to IBV in Indonesia is still limited to poultry. Studies of diseases on endemic species are valuable for the conservation effort, yet are rarely conducted.

The Eclectus parrot (*Eclectus roratus*) is a sexually dichromatic parrot native to a part of Eastern Indonesia and Northern Australia [11]. It is classified as protected in Indonesia according to Government Decree Number 7, Year 1999 and Constitution Number 5, Year 1990. Visually captivating, with both male and female showing radically different plumage, the Eclectus parrot is naturally talkative and popular as a pet [11]. However, there has been limited information about viral diseases among Eclectus parrots. The latest finding on coronavirus in parrots was in 2006 when a virus distinct from IBV was found in the green-cheeked Amazon parrot [12]. Understanding viral diseases in Eclectus parrots may be beneficial for the conservation effort and may provide additional information about viral diseases in birds.

There is limited information as to whether avian coronaviruses cause diseases in Psittacine birds; therefore, information about the presence of this virus among parrots might be valuable for the conservation effort of endemic birds and the poultry industry, which is robust in Indonesia. This study aimed to determine the presence of and to characterize avian coronavirus isolated from Eclectus parrots reared by an Indonesian local bird breeder.

## Materials and Methods

### Ethical approval

The methods performed in this research have been approved by the Ethical Committee of the Faculty of Veterinary Medicine, Bogor Agricultural University, Indonesia, which were validated with the certificate number 058/KEH/SKE/IV/2017.

### Samples

Cloacal swab samples were obtained from 10 healthy and clinically sound Eclectus parrots (*E. roratus*), which belong to a bird breeder in Bogor, West Java, Indonesia. Swabs were stored in viral transport media containing phosphate-buffered saline, penicillin (1000 IU/ml), and streptomycin (10 mg/ml). Samples were carried in a cooler box during transportation and stored at −80°C until use. All birds had never been vaccinated. The IBV vaccine strain H120 was used as a positive control.

### Virus isolation

All isolates were propagated into three specific pathogen-free (SPF) embryonated chicken eggs. The swab suspension was mixed with antibiotics (penicillin 1000 IU/ml and streptomycin 10 mg/ml) in a 4:1 v/v ratio. A 0.2 ml swab suspension was inoculated through the allantoic route into a 9-day-old SPF embryonated chicken eggs and then incubated at 36°C for 4 days or until the embryo died. Allantoic fluid was harvested on day 4 post-inoculation and stored at −20°C until use.

### Viral RNA extraction

Total viral RNA was extracted using the RNeasy Mini Kit (Qiagen, Germany) according to the manufacturer’s instructions. The RNA was dissolved in 30 µl RNase-free water and used for amplification by reverse transcriptase-polymerase chain reaction (RT-PCR). The RNA was stored at −20°C until use.

### Amplification

RT-PCR was performed with the following primer pairs, as described in Table-1: UTR41+/11− targeting the 3’ untranslated region (3’-UTR) of the avian coronavirus genome [13], IBVN+/IBVN− targeting the N gene of IB [14], and XCE2+/XCE2− targeting the S1 gene [15]. Polymerase chain reaction amplification was carried out using the Bioline One-Step RT-PCR kit (Bioline, UK) with the following thermal cycling profile: Reverse transcription at 45°C for 20 min, polymerase activation at 95°C for 1 min, then 40 cycles of denaturation at 95°C for 15 s, annealing temperatures and times specific for each primer pair and described below, and extension at 72°C for 30 s. This was followed by a final extension at 72°C for 7 min. Annealing occurred at 48°C for 1 min, 58°C for 30 s, and 50°C for 30 s for UTR41+/11− [13], the partial N gene [14], and the partial S1 gene [15], respectively.

**Table-1:** Nucleotide sequences of primers used.

Primer	Sequence	Length	Source
UTR41+	5’- ATGTCTATCGCCAGGGAAATGTC-3’	266 bp	[13]
UTR11−	5’- GCTCTAACTCTATACTAGCCTA -3’	266 bp	[13]
IBVN+	5’- GAAGAAAACCAGTCCCAGATGCTTGG-3’	453 bp	[14]
IBVN−	5’- GTTGGAATAGTGCGCTTGCAATACCG-3’	453 bp	[14]
XCE2+	5’- CAC TGG TAA TTT TTC AGA TGG-3’	466 bp	[15]
XCE2−	5’- CCTC TAT AAA CAC CCT TACA-3’	466 bp	[15]

### Nucleotide sequencing

Following amplification using the partial S1 gene primer XCE2−/XCE2+ pairs, 30 µl of the nucleotide sample was sent to First Base (Malaysia) to be sequenced from both directions. The resulting nucleotide sequences were aligned and compared with the S1 gene available from GenBank. Nucleotides taken from GenBank were used for alignment and tree construction, including genes that represent each IBV lineage according to Valastro *et al*. [16], Indonesian isolates from Wibowo *et al*. [17], and LX4 (AY189157) and Israel variant 1 (AF0937940), to illustrate well-used QX and 4/91 variant sequences. The genes used in the analysis are listed in Table-2. A phylogenetic tree was constructed using the maximum likelihood method using MEGA 7 software (Pennsylvania, USA) with 1000 bootstrap replications. Nucleotide and amino acid distances were calculated and provided for the following genes: H120 vaccine as a positive control (FJ888351), peafowl/GD/KQ6/2003 (AY641576) as a non-chicken IBV-like virus, MHW-QX-MGX-2012 (MH671339) as an Indonesian Qx-type sample, MHW-O-NSTR-2-2018 (MH671342) as an Indonesian 4/91 sample, Moroccan G/83 (EU914938) as a GI-13 lineage representative, 4/91 Israel variant 1 (AF093794), 4/91 vaccine (KF377577), and the isolate, parrot/Indonesia/BX9/2016.

**Table-2:** List of sequences used in phylogenetic analysis.

Strain (isolate name)	Lineage	Country	Collection year	GenBank accession no.
Beaudette	GI-1	USA	1937	M95169.1
Holte	GI-2	USA	1954	GU393336.1
Gray	GI-3	USA	1960	L14069.1
Holte	GI-4	USA	1962	L18988.1
N1/62	GI-5	Australia	1962	U29522.1
VicS	GI-6	Australia	1962	U29519.1
TP/64	GI-7	Taiwan	1964	AY606320.1
L165	GI-8	USA	1965	JQ964061.1
ARK99	GI-9	USA	1973	M99482.1
B	GI-10	New Zealand	1970	AF151954.1
UFMG/G	GI-11	Brazil	1975	JX182775.1
D3896	GI-12	The Netherlands	1978	X52084.1
Moroccan-G/83	GI-13	Morocco	1983	EU914938.1
B1648	GI-14	Belgium	1984	X87238.1
B4	GI-15	Korea	1986	FJ807932.1
IZO 28/86	GI-16	Italy	1986	KJ941019.1
CA/Machado/880	GI-17	USA	1988	AF419315.1
JP8127	GI-18	Japan	1993	AY296744.1
58HeN-93II	GI-19	China	1993	KC577395.1
Qu_mv	GI-20	Canada	1996	AF349621.1
Spain/97/314	GI-21	Spain	1997	DQ064806.1
40GDGZ-971	GI-22	China	1997	KC577382.1
Variant 2	GI-23	Israel	1998	AF093796.1
V13	GI-24	India	1998	KF757447.1
CA/1737/04	GI-25	USA	2004	EU925393.1
NGA/B401/2006	GI-26	Nigeria	2006	FN182243.1
GA08	GI-27	USA	2008	GU301925.1
D1466	GII-1	The Netherlands	1979	M21971.1
N1/88	GIII-1	Australia	1988	U29450.1
DE/072/92	GIV-1	USA	1992	U77298.1
N4/02	GV-1	Australia	2002	DQ059618.1
TC07-2	GVI-1	China	2007	GQ265948.1
H120 vaccine	GI-1*	Indonesia	2017	Not registered
4/91 vaccine	GI-13*	Indonesia	2017	Not registered
4/91 Israel variant 1	GI-13*	Israel	1998	AF093794.1
4/91 (Chicken/Attock/MARC-786/2013)	GI-13*	Pakistan	2013	KU145467.1
4/91 (CK/CH/YN/SL 1301-1)	GI-13*	China	2016	KX107779.1
4/91 (gammaCoV/Ck/Poland/G193/2015)	GI-13*	Poland	2015	MK576138.1
4/91 (MHW-Lay-Mikro-2017)	GI-13*	Indonesia	2017	MH671335.1
4/91 (MHW-Kodil-2017)	GI-13*	Indonesia	2017	MH671337.1
4/91 (MHW-Solo-Lay-2017)	GI-13*	Indonesia	2017	MH671336.1
4/91 (MHW-O-NSTR-2-2018)	GI-13*	Indonesia	2018	MH671342.1
LX4	GI-19	China	2002	AY189157.1
Qx-like (MHW-QX-MGX-1-2012)	GI-19*	Indonesia	2012	MH671339.1
Qx-like (MHW-QX-KDL-3-2012)	GI-19*	Indonesia	2012	MH671340.1
Qx-like (MHW-Rhb-5-2017)	GI-19*	Indonesia	2017	MH671338.1
Indonesia/K233A31/18	GI-13*	Indonesia	2018	Not registered
Indonesia/K4A9/17	GI-13*	Indonesia	2017	Not registered
Indonesia/P3/17	GI-13*	Indonesia	2017	Not registered

**Figure-1:**
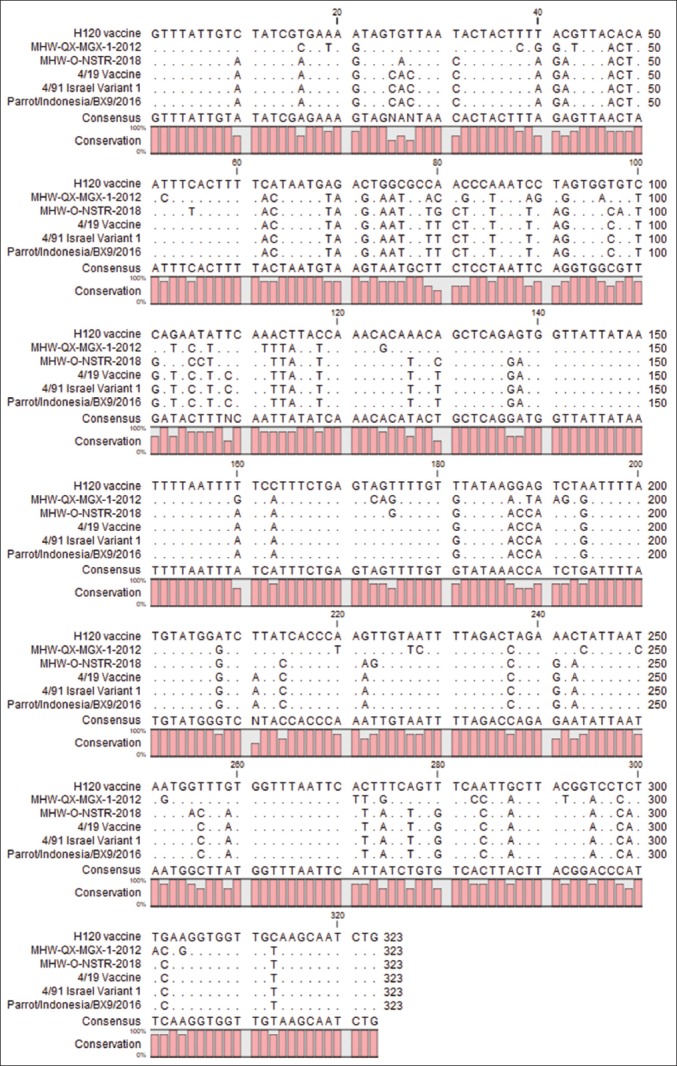
Alignment of sample isolate (Parrot/Indonesia/BX9/2016) from the Eclectus parrot with the H120 vaccine positive control (FJ888351.1), the 4/91 vaccine strain (KF377577.1), the Indonesian isolate (MH671341.1 for the 4/91-like isolate and MH671339.1 for the Qx-like isolate), and the 4/91 Israel variant 1 (AF093794.1) strain.

* According to phylogenetic tree generated in Figure 1

## Results

### Isolation and identification of avian coronavirus

Amplification was performed on original swab samples and allantoic fluids. None of these samples was positive for the 3’-UTR of the avian coronavirus (UTR11−/41+). Three out of 10 swab samples were positive for the N gene coronavirus, but none of them was positive for the S1 IBV gene. However, four out of 10 samples of allantoic fluid tested were positive for the coronavirus N gene. Of the two that tested positive for the S1 gene of IBV, only one of them, which was designated as parrot/Indonesia/BX9/16, was able to be further sequenced. These results are presented in Table-3.

**Table-3:** The result of reverse transcriptase-polymerase chain reaction amplification for each isolate.

Isolate	Swab			Allantoic fluid		
	
	3’UTR	N	S1	3’UTR	N	S1
Parrot/Indonesia/BX1/16	−	+	−	−	−	−
Parrot/Indonesia/BX2/16	−	−	−	−	−	−
Parrot/Indonesia/BX3/16	−	−	−	−	−	−
Parrot/Indonesia/BX4/16	−	−	−	−	+	−
Parrot/Indonesia/BX5/16	−	−	−	−	−	−
Parrot/Indonesia/BX6/16	−	−	−	−	−	−
Parrot/Indonesia/BX7/16	−	+	−	−	+	+
Parrot/Indonesia/BX8/16	−	+	−	−	+	−
Parrot/Indonesia/BX9/16	−	−	−	−	+	+
Parrot/Indonesia/BX10/16	−	−	−	−	−	−

### Nucleotide sequencing of the partial S1 gene

**Figure-2:**
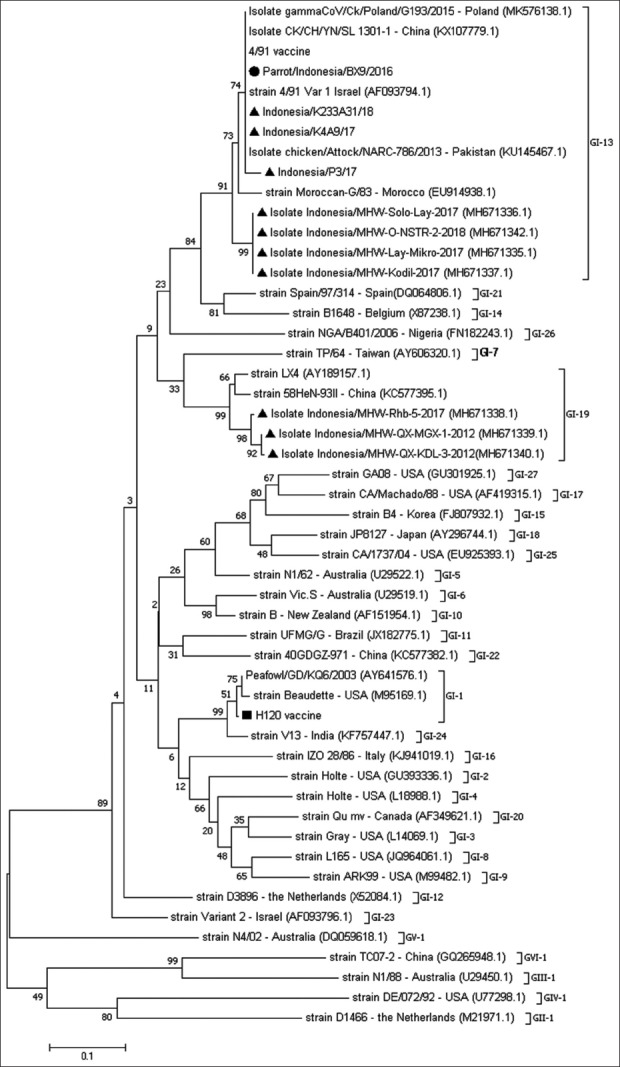
Phylogenetic tree constructed using the maximum likelihood method with 1000 bootstrap replicates. Sample isolate Parrot/Indonesia/BX9/2016 is marked with (•). Positive control H120 vaccine is marked with (■). Indonesian isolates are marked with (▲).

Only one isolate, defined as parrot/Indonesia/BX9/16, was sequenced for the partial S1 gene of IBV using XCE2+/XCE2− primers (Table-1). Nucleotide sequencing of 323 nucleotides from the partial S1 gene showed that there was no difference in the nucleotide sequence of the parrot/Indonesia/BX9/16 gene when compared with IBV 4/91 Israel variant 1 (AF093794.1) and the 4/91 vaccine strain (KF377577.1) (Figure-1). The nucleotide and amino acid pairwise distance also showed 100% homology with the IBV 4/91 Israel variant 1 (AF093794.1) and the 4/91 vaccine strain (KF377577.1). However, differences were observed between the sequenced gene, the H120 (FJ888351) positive control, and the non-chicken IBV-like peafowl/GD/KQ6/2003 virus (AY641576) (Table-4). A phylogenetic tree (Figure-2) of the aligned nucleotide sequence of the partial S1 gene was constructed using the maximum likelihood method with Mega 7 software with 1000 bootstrap value. The tree showed a close relatedness of viral isolate, parrot/Indonesia/BX9/16, to the IBV strain 4/91 variant 1 Israel (AF093794.1), the 4/91 vaccine strain (KF377577.1), CK/CH/YN/SL 1301-1 (KX107779.1), chicken/Attock/NARC-786/2013 (KU145467.1), and gammaCoV/Ck/Poland/G193/2015 (MK576138.1), whereas there were differences observed when compared with the H120 vaccine (FJ888351.1) positive control.

**Table-4:** Alignment of nucleotide and amino acid sequences between parrot/Indonesia/BX9/16, positive control and samples from Indonesian isolate and 4/91 strains obtained from GenBank.

Samples	1	2	3	4	5	6	7	8

	Nucleotide and amino acid identity							
H120_vaccine (FJ888351.1)		0.009	0.186	0.194	0.203	0.190	0.190	0.190
Peafowl/GD/KQ6/2003 (AY641576.1)	0.013		0.179	0.182	0.194	0.178	0.178	0.178
MHW-QX-MGX-2012 (MH671339.1)	0.511	0.532		0.155	0.161	0.152	0.152	0.152
MHW-O-NSTR-2-2018 (MH671342.1)	0.693	0.668	0.575		0.044	0.029	0.029	0.029
Moroccan-G/83 (EU914938.1)	0.693	0.668	0.575	0.163		0.027	0.027	0.027
4/91_vaccine_(KF377577.1)	0.668	0.644	0.553	0.148	0.092		0.000	0.000
4/91_Israel_Variant_1 (AF093794.1)	0.668	0.644	0.553	0.148	0.092	0.000		0.000
Parrot/Indonesia/BX9/2016	0.668	0.644	0.553	0.148	0.092	0.000	0.000	

## Discussion

IBV and IB-like viruses, which are avian coronaviruses, have been detected among avian species other than chickens, such as pheasants, peafowl, turkeys, teal, pigeons, geese, penguins, quail, ducks, waterfowl, and Amazon parrots [2,9,10]. Advances in genetic identification by biomolecular technologies allow rapid identification of viruses without the need for prior isolation. However, the question remains as to whether these novel viruses cause disease in the species that they were found [8,3]. There have been few instances where isolates were obtained, such as a virus that was found in peafowl that was found to share >99% identity with H120 IBV vaccines [18] and Mass-type IBV [19]. A coronavirus detected in pigeons was found to be clustered as a Mass-type IBV [20], whereas a coronavirus found in teal has been shown to exhibit >80% genetic similarity with several IBV strains including infectious strains [21]. Interestingly, upon exposure to chickens, the peafowl IB-like virus, which is close to IBV vaccine strain H120, did not cause disease among these birds [18], whereas the IB-like Mass-type peafowl isolate, the IB-like Mass-type pigeon isolate, and the IB-like teal isolate caused infection in chickens [19,20,21]. These previous studies indicate that the IB-like virus may be found in hosts other than chickens and that they might originate from the chicken IBV or might be a novel virus. However, unless this is investigated further, it is uncertain whether the viruses found in other species of birds can cause disease in chickens.

The S1 gene of IBV is often used for genotyping. It is important in determining attachment and houses most of the virus epitopes, which can affect the virus serotypes [2,8,7]. The nucleotide sequence of the partial S1 gene used in this study (XCE2+/XCE2−) only covers the hypervariable region (HVR)-3 of the S1 gene [22] but does not cover HVR 1 and HVR 2. This present study indicated that within the section of the parrot/Indonesia/BX9/16 gene that was sequenced, there was 100% similarity with several IBV 4/91 strains taken from GenBank, which included both infectious and vaccine strains, namely: IBV strain 4/91 variant 1 Israel (AF093794.1), 4/91 vaccine strain (KF377577.1), CK/CH/YN/SL 1301-1 (KX107779.1), chicken/Attock/NARC-786/2013 (KU145467.1), and gammaCoV/Ck/Poland/G193/2015 (MK576138.1). Recent publications regarding IBV in Indonesia by Wibowo *et al*. [17] and Setiawaty *et al*. [22] showed the presence of a circulating infectious 4/91 strain in Indonesia. Sequences obtained from Wibowo *et al*. [17] were 4/91-like IBV originating from Indonesia in 2017 to 2018. These sequences were included in our phylogenetic analysis but appeared to be slightly different from our isolated virus in each of the nucleotides numbered: 55, 80, 98, 175, 211, 223, and 225. However, two sequences from Setiawaty *et al*. [22] showed 100% similarity (Indonesia/K233A31/18 and Indonesia/K4A9/17) to our isolated gene and only one slight variation compared with their Indonesia/P3/17 gene. The study by Setiawaty *et al*. [22] showed that the 4/91 gene recovered from an outbreak may show sequence homology to the live vaccine. The study by Wibowo *et al*. [17] was conducted in East Java, while the study by Setiawaty *et al*. [22] was conducted in West Java, which is in close proximity to the place that this research was conducted. This might explain why, in our study, we were able to isolate an IB-like virus that was close in homology to the IBV 4/91 strain. This also indicates that the 4/91 strain is widespread in Java Island, Indonesia.

According to Valastro *et al*. [16], the IBV 4/91 strain and 793.B belong to the IBV GI-13 lineage. This lineage is among the most widely distributed lineage in the world. This has mostly contributed to the widespread usage of vaccinations [16]. However, in Indonesia, vaccines commonly used in poultry are from the GI-1 lineage, which consists of Mass-type IBV such as M41, H52, or H120 strains [17,22]. Information dating back to 1977 and 1985 states that at that time, circulating IBV in Indonesia was close to the IBV Massachusetts, IBV Connecticut, and Australian strains [23]. The IBV variant strain was first detected in Indonesia in 2003 [24]. A recent study of circulating IBV in outbreaks among chickens in Indonesia showed that the virus isolated in 2012 in East Java was Qx-like and was similar to China and Taiwan strains in 2017 [25]; whereas, IBV isolated in 2018 was found to be close in similarity to the IB 4/91 strain [17,22]. In general, IBV strains within the same serotype share >95% amino acid similarity and those with <85% similarity usually belong to different strains [2]. A slight change in the S1 gene may affect the serotype of the virus and cross-protection [2,6]. This could probably explain why our virus was similar to vaccine and field viruses at the same time. Despite the viruses having 100% homology on the site we sequenced, differences in other parts of the genome may cause differences in virulence. Considering how 100% homology was obtained with both the vaccine virus and field strains, it is difficult to determine the origin or infectivity of the virus found. The birds sampled in this research were from a breeding institution without any vaccination program; thus, the virus found did not develop from any attempted vaccination program to said captive birds. The birds sampled appeared to be healthy, but it is unknown whether the isolated virus can cause infection in chickens. The birds were also born and bred in the same institution, so the path of infection is unknown. Further research on biosecurity, including factors such as water quality or the possibility of contact with wild birds, is needed to identify the origin of infection.

Evidence of infections by the IBV vaccine virus or the infectious IBV strain to birds of other species has been reported before. Reverse spillover of the vaccine virus from domesticated birds to wild birds has been proposed by Devlin *et al*. [5] and Rohaim *et al*. [26]. Our results were similar to the previous study by Ito *et al*. [18], which discovered that IB-like coronavirus from peafowl has close genetic relatedness with the IBV H120 vaccine strain. This previous study also showed that the vaccine strain may infect non-chicken birds and may have few mutations that, upon infection to chickens, do not inflict disease. Liu *et al*. [21] have also suggested that not only the vaccine strains but also a possible infectious IBV strain may be transmitted from poultry to birds of other species. For example, cross-transmission of nephropathogenic IBV virus occurred in teal housed near domesticated poultry [21]. This means that IBV can infect a variety of bird species. In a possible case where infected birds are wild birds, these birds may act as a reservoir of the virus and spread it to other susceptible species [27]. Transmission of the virus to wild birds has likely happened due to poor biosecurity in poultry farms and contamination from improper disposal of litter and waste [5]. Most of the Indonesian poultry farmers still rely on an Open House Coop. A high density of birds without effective biosecurity and disease prevention increases the possibility of viral mutation [8]. Contamination from improper disposal of litter and waste may ease the transmission from domesticated poultry to wild birds. Spillover to wild birds is often overlooked, especially in terms of its ecological impact. This is especially important for endangered species. However, the epidemiology and effect of spillover from domesticated birds to wild birds are not yet fully understood, despite evidence of its occurrence.

Not only impacting wild birds but also this spillover effect is important for the poultry industry. Other RNA avian viruses with similarly high mutation rates such as avian influenza (AI) and Newcastle disease virus (NDV) illustrate the importance of disease transmission between poultry and wild birds. The emergence and spread of highly pathogenic AI often contribute to its presence in wild birds [28,29]. In the case of AI, mutation and recombination possibly happened in wild birds and emerged as a highly pathogenic virus in poultry to a devastating effect. Its spread to various continents might also be attributed to migrating waterfowl carrying the virus [29]. Persistent outbreaks, despite a rigorous vaccination effort, such as what has happened in IBV, can be seen in NDV [30]. Studies on both free-living birds and poultry show how NDV has circulated and continued to evolve in the environment [30,31]. These cases, although they describe other viruses, illustrate the importance of biosecurity and surveillance to prevent possible emerging virulent viruses in the future through the transmission of disease between free-living birds and domestic birds. This is especially important for developing countries where many still practice backyard farming and small-scale farming with low biosecurity [28,31], such as those in Indonesia. Unfortunately, interspecies studies of IBV are rarely conducted in the country. This study is the first to have studied IBV in a species of bird other than chicken in Indonesia.

The similarity between viruses isolated from captive birds and domesticated poultry, as found in this research, is alarming, as there is an indication of a reverse spillover effect that has already happened from poultry farms to the environment. Our findings also indicated that IBV has a broad range of hosts, opening more possibility of a spillover effect to happen on environmental contamination. This finding was in accordance with the previous study that isolated avian coronavirus in waterfowls and teal that did not show any symptoms of the disease [19,21]. There is a possibility that wild birds and other non-galliform birds may harbor IBV or IB-like coronavirus without showing any symptoms and illustrate the wide host range of coronavirus.

## Conclusion

IB-like coronavirus isolated from the Eclectus parrot from an Indonesian bird breeder has 100% homology with IBV 4/91 variant 1 Israel, a 4/91 vaccine strain, CK/CH/YN/SL 1301-1, chicken/Attock/NARC-786/2013, and gammaCoV/Ck/Poland/G193/2015. IB-like coronavirus was isolated from healthy parrots, indicating that this bird might act as a reservoir for coronavirus.

## Authors’ Contributions

GKS performed the work. EH, RDS, AS, and ONP contributed to designing the study and method. All authors contributed to the writing and revision of the manuscript. All authors read and approved the final manuscript.
